# Characterization of Microbial Communities, Identification of Cr(VI) Reducing Bacteria in Constructed Wetland and Cr(VI) Removal Ability of *Bacillus cereus*

**DOI:** 10.1038/s41598-019-49333-4

**Published:** 2019-09-06

**Authors:** Hua Lin, Shaohong You, Liheng Liu

**Affiliations:** 1 Guangxi Key Laboratory of Environmental Pollution Control Theory and Technology, Guilin University of Technology, Guilin, China; 2 Collaborative Innovation Center for Water Pollution Control and Water Safety in Karst Area, Guilin University of Technology, Guilin, China

**Keywords:** High-throughput screening, Pollution remediation

## Abstract

In this study, the contribution of substrates microorganisms in three different constructed wetlands (CWs) to Cr(VI) purification was discussed. In addition, the microbial communities in the substrate of different CWs were characterized, and rhizosphere Cr(VI) reducing bacteria was also identified. The results showed that microorganisms could improved Cr(VI) removal to 76.5%, and result in that more Cr(VI) was reduced to Cr(III). The dominant strains in the substrates of different CWs were *Sphingomonas* sp., *Cystobacter* sp., *Acidobacteria bacterium*, *Sporotrichum* and *Pellicularia species*. The Cr(VI) reducing bacteria from *Leersia hexandra Swartz* rhizosphere was identified as *Bacillus cereus*. Furthermore, under suitable conditions, the removal rate of Cr(VI) by *Bacillus cereus* was close to 100%.

## Introduction

Due to the strong aqueous solubility, bioavailability and persistence^1^, Cr(VI) has become one of the most noxious metals to the water resources^2^. Therefore, it is necessary to remove Cr(VI) from water resources for environmental safety and human health. In China, reduction precipitation, electrochemistry and ion exchange are widely used in the treatment of wastewater containing Cr(VI). However, they also have obvious shortcomings such as high sludge production, high energy consumption, and difficulty in operation and maintenance. Moreover, the Cr(VI) content of the effluent of these technologies is no longer able to meet the new emission standards. It is necessary to develop new technologies for wastewater containing Cr(VI) treatment.

In recent years, various techniques such as adsorption, electrocoagulation, membrane separation, photocatalysis, and constructed wetlands have been applied to the treatment of wastewater containing water^3,4^. Comparing with other methods, constructed wetlands (CWs) are widely used in the treatment of water resources containing Cr(VI), because of the ecological and environmental advantages, and economic and social benefits^5^. Therefore, CWs has become one of the hotspots in the treatment of wastewater containing Cr(VI)^2^.

The Cr(VI) is removed by reduction, precipitation, adsorption, filtration, plant uptake, complexation and precipitation in CWs^6^. The plants, substrate and microorganisms in CWs which are interactive all play important roles in Cr(VI) removal process^7^. In CWs, the contribution of plants to Cr(VI) removal is mainly by the accumulation in the root tissues, or by the *in situ* detoxification and reduction, or by oxidation^8^. Compared to plant-free constructed wetlands, plants can significantly improve the removal efficiency of Cr(VI)^2^. However, when the concentration of Cr(VI) in water is high, Cr(VI) inhibits plant growth^9^. Therefore, the application of Cr super-enriched plants to remove Cr(VI) in CWs is more concerned. *Leersia hexandra Swartz* is a kind of Cr super-enriched plant widely distributed in China^10^. Our previous studies have shown that *Leersia hexandra Swartz* CWs has good removal of Cr(VI) in water even when the concentration of Cr(VI) in water is 7.50 mg/L^6,7^. However, our further research indicates that more than 90% of Cr(VI) is immobilized in the substrates by substrates and microorganisms, and the Cr(VI) directly removed by *Leersia hexandra Swartz* is less than 10%. This suggests that the substrates and microorganisms are the key to removing Cr(VI) from constructed wetlands.

A suitable substrate not only could remove Cr(VI) by adsorption or ion exchange^11,12^, but also could increase the permeability and hydraulic load of the CWs^13^. At present, biochar used as substrate has attracted extensive attention because of its enhancement of soil fertility, attraction of beneficial microorganisms, and improvement of pollutants removal^14,15^. For example, the addition of biochar to the substrate can significantly increase the abundance of *Proteobacteria*, *Acidobacteria*, *Firmicutes* and *Actinobacteria*, and promote the transformation of the dominant species from *Rudeea* to *Bacillus* which has strong degradation ability to chlorpyrifos^16^. The contribution of microorganisms to Cr(VI) removal is achieved through biosorption and bioreduction^17,18^. Singh *et al*.^19^ confirmed that inoculation of Cr-reducing bacteria at the root of plants can increase the removal of Cr(VI) by constructed wetlands. Moreover, microorganisms can also form symbionts with plant roots, thereby increasing plant uptake of heavy metals^20^. Unfortunately, most researches pay attention on the effect of plants and matrix on Cr(VI) removal^4,21,22^, while there are a few reports about the effect of microorganisms on Cr(VI) removal^9,23^.

In this study, three different CWs were used to treat Cr(VI) contaminated water for three special purposes. First, the contribution and role of microorganisms in CWs for the Cr(VI) removal were determined. Second, the characteristics of microbial communities in substrate were investigated based on the results of polymerase chain reaction-denaturing gradient gel electrophoresis (PCR-DGGE). Last, the Cr(VI)-reducing bacteria in the plants rhizosphere were isolated and identified, and its ability of reducing Cr(VI) was also discussed.

## Materials and Methods

### Materials

The soil used as the substrate of the constructed wetland was collected from the surface of the beach (0–20 cm) at the junction of the Lijiang River and the Liangfeng River. After the soil is naturally air-dried, it passes through a 60 mesh sieve. The bagasse biochar, also used as a substrate, was prepared by a two-step pyrolysis process at 200 °C and 500 °C, respectively. The bagasse biochar also passed through a 60 mesh sieve. The physical and chemical properties of soil and bagasse biocahr are shown in Table 1.

**Table 1:** Physical and chemical properties of the substrates.

Substrates	pH	Ca (mg/kg)	Mg (mg/kg)	K (mg/kg)	Cr (mg/kg)	Cr(VI) (mg/kg)	Organic carbon (g/kg)	Effective phosphorus (g/kg)
Soil	6.32	15120.35	1235.55	256.55	5.07	ND	35.67	18.49
Bagasse biochar	8.62	18280.46	1790.22	3217.06	ND	ND	332.50	952.12

The Cr(VI)-containing wastewater was prepared from potassium dichromate (analytical grade) and tap water. The Cr(VI) concentrations were 5.00 mg/L and 25 mg/L, respectively.

*Leersia hexandra Swartz* was collected from the non-polluted area of the Liangfeng River beach in Yanshan District, Guilin, China. It was trimmed to 15 cm and then washed with pure water. The trimmed *Leersia hexandra Swartz* was incubated in Hoagland nutrient solution for 5 d to restore the damaged roots during transport.

### Design and operation of constructed wetlands

**Figure 1:**
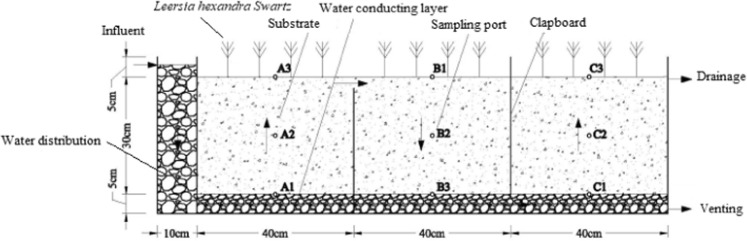
Schematic diagram of the three-stage wavy subsurface flow constructed wetland.

In this study, a three-stage wavy subsurface flow constructed wetland was designed, the details of which are shown in Fig. 1. On this basis, according to the experimental purpose, three different forms of wetlands, 1# CWs, 2# CWs and 3# CWs, were designed and operated. The details of these three wetlands are shown in Table 2. After 156 d of operation, the substrates of the three CWs were sampled using a quincunx method. The samples were freed of impurities and stored aseptically sealed at 4 °C.

**Table 2:** The parameters of construction and operation of different constructed wetlands^7^.

CWs No.	Plants	Substrate type and mass ratio	Influent flow (m^3^/d)	Cr (VI) concentration (mg/L)
1# CWs	Leersia hexandra Swartz	bagasse biochar and soil; 5:95	0.06	5
2# CWs	Leersia hexandra Swartz	bagasse biochar and soil; 5:95	0.06	0
3# CWs	None	bagasse biochar and soil; 5:95	0.06	5

### Purification of Cr(VI) by microorganisms in substrates

The substrate sample collected from 1# CWs was divided into three groups for different treatments. The first group of samples did not take any treatment, while the second group and the third group of samples were sterilized by high-temperature autoclaving and chloroform sterilization, respectively.

Before usage, the potassium dichromate solution with Cr(VI) concentration of 25 mg/L was first filtered with a 0.22 micron membrane to remove bacteria. The differently treated substrates with the quality of 10 g were separately added to three 150 mL Erlenmeyer flasks, and then 50 mL of the filtered solution was added to the Erlenmeyer flasks. The Erlenmeyer flasks were shaken at 150 rpm at 30 °C. After 72 h, the mixtures in the Erlenmeyer flasks were transferred to three 10 mL centrifuge tubes, respectively, and centrifuged at 4000 rpm for 10 min. The Cr(VI) and total Cr concentrations in the supernatant samples were determined by diphenylcarbazide spectrophotometry and potassium permanganate oxidation-diphenylcarbazide spectrophotometry, respectively. The Cr(VI) and total Cr in the solid phase were extracted by basic digestion method (EPA3060A) and acidic digestion method, respectively, and their concentrations were also determined by diphenylcarbazide spectrophotometry and potassium permanganate oxidation-diphenylcarbazide spectrophotometry, respectively. The concentration of Cr(III) is the difference between total Cr and Cr(VI).

### Analysis of microbial communities in substrates

According to the manufacturer’s instructions, the total DNA of the substrate sample was extracted using the Powersoil soil DNA extraction kit (MoBio Laboratories, Carlsbad, US). For bacterial communities, two rounds of 16S rDNA polymerase chain reaction (PCR) amplifications of the V3 region were performed. The amplification primers were universal primers 27F/1492R (5′-AGA GTT TGA TCC TGG CTC AG-3′/5′-TAC GAC TTA ACC CCA ATC GC-3′) and primers 357F/518R(5′-CCT ACG GGA GGC AGC AG-3′/5′-ATT ACC GCG GCT GCT GG-3′)^24,25^, respectively. The temperature cycling conditions of PCR were as follow: 94 °C for 5 min; 30 cycles (94 °C for 30 s; 56 °C for 30 s; 72 °C for 1.5 min (27F/1492R)/30 s (F357-GC/518R)), and 72 °C for 10 min.

Fungal communities were characterized by nested PCR amplification using primers ITS1/ITS4 (5′-TCC GTA GGT GAA CCT GCG G-3′/5′-TCC GTA GGT GAA CCT GCG G-3′) and ITS1GC/ITS2 (5′-CGC CCG CCG CGC GCG GCG GGC GGG GCG GGG GCA CGG GGG G TCC GTA GGT GAA CCT GCG G-3′/5′-GCT GCG TTC TTC ATCG ATG C-3′) as the first and second round primers, respectively^26,27^. The temperature cycling conditions of nested PCR were as follows: 94 °C for 5 min, 35 cycles (94 °C for 30 s; 50 °C (ITS1/ITS4) or 56 °C (ITS1GC/ITS2) for 30 s; 72 °C for 1 min), and 72 °C for 10 min.

The PCR products were separated by DGGE on 8% polyacrylamide gels in 1 × TAE buffer, and detected by the D-Code Mutation Detection System (Bio-Rad, US)^19^. The linear denaturing gradients for bacteria and fungi were 35~65% and 30~50%, respectively. Electrophoreses were performed at 70 V and 60 °C for 14 h. Then, the gels were rinsed with ultrapure water and stained with a dye bath containing 1% Goldview. The Quantity One software was used to identify the lanes and bands of the DGGE profiles. Basing on the results of Quantity One, the coefficient of similarity (Cs) and diversity index (Di) were calculated.

The representative bands were excised and crushed. After elution with 50 μL sterile distillation-distillation H_2_O, they were stored overnight at 4 °C. The 1 μL soaking solution was selected as a template and amplified using primers 357F/518R. The PCR products were sequenced with primer F357 by Invitrogen Biotechnology Co. Ltd. (Shanghai, China). And the sequence was submitted to the NCBI database for BLAST alignment.

### Isolation and identification of rhizosphere Cr(VI) reducing bacteria

Liquid Luria-Bertani culture and solid Luria-Bertani culture were used to separate and culture Cr(VI) reducing bacteria. Their components were as follows: peptone 10 g/L, yeast extract powder 5 g/L, NaCl 5 g/L (liquid Luria-Bertani culture); peptone 10 g/L, yeast extract powder 5 g/L, NaCl 5 g/L, 1.5% agar (solid Luria-Bertani culture). They were sterilized at 121 °C for 20 min.

The soil collected from the rhizosphere of Leersia hexandra Swartz was cultured in liquid Luria-Bertani culture for 12 h at 37 °C. Then, the supernatant was transferred to a liquid Luria-Bertani culture containing 5 mmol of Cr(VI), and also cultured at 37 °C for 12 h. In order to screen out the Cr(VI) reduced strain, the bacteria enriched in liquid Luria-Bertani culture were transferred to solid Luria-Bertani culture and cultured for 48 h at 37 °C^28^.

The isolated strain was first rejuvenated and cultured for 35 h. After this, the DNA of the strain was extracted and subjected to 16S rDNA PCR amplification. The primers of PCR amplification were 5′-AGA GTT TGA TCC TGG CTC AG-3′ (16 s rDNA forward primer 27F), 5′-ACG GTT ACC TTG TTA CGA CTT-3′ (16 s rDNA reverse primer 1492R)^29^, 5′-AAAgTTTAAAgAAgTACAAgAAgC-3′ (dnaJ gene forward primer) and 5′-CTTTACCATgAACAgTAggAAC-3′ (dnaJ gene reverse primer). And the 16 s rDNA PCR reaction conditions and dnaJ gene sequence reaction conditions are as follows: 95 °C for 300 s, 95 °C for 60 s, 57 °C for 60 s, 72 °C for 80 s, 30 cycles, 72 °C for 300 s, 4 °C preservation (16 s rDNA PCR); 95 °C for 300 s, 32 cycles of 94 °C for 40 s, 48 °C for 40 s, and 72 °C for 60 s, 72 °C for 300 s, storage at 4 °C (dnaJ gene sequence). Finally, Biolog GENIIIGEN III Micro Plate^30^ and API 20E reagent strips^31^ were used to test the physiological and biochemical characteristics of Cr(VI) reducing strain according to the manufacturer’s instructions.

### Test of reducing ability of Cr(VI) reducing bacteria

The effects of Cr(VI) initial concentration, initial pH value, reaction temperature and inoculation amount on the reducing ability of Cr(VI) reducing bacteria in rhizosphere were investigated. A certain amount of K_2_Cr_2_O_7_ was added to the liquid Luria-Bertani cultures in which the Cr(VI) reducing bacteria inoculations were 1%, 5%, 10%, 15%, and 20%, respectively, so that the concentration of Cr(VI) was in the range of 20 to 100 mg/L. The pH of the liquid medium was adjusted in the range of 5–9, while the culture temperature range was 25~45 °C. The Cr(VI) concentration of supernatant was measured by diphenylcarbazide spectrophotometry every 12 h.

## Results and Discussion

### Contribution of microorganisms to Cr(VI) purification

**Figure 2:**
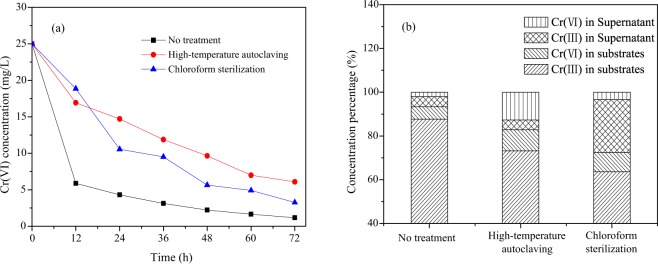
Cr(VI) concentrations in substrates (**a**) and chromium mass balance (**b**).

The contribution of substrates microorganisms to Cr(VI) purification is shown in Fig. 2. Form Fig. 2(a), it can be seen that the contribution of microorganisms to Cr(VI) purification is great, especially in the early stages of Cr(VI) removal. When the time was 12 h, the removal rate of Cr(VI) was 76.5% in untreated substrate, which was 2.36 and 3.18 times that of high-temperature autoclaving and chloroform sterilization, respectively. According to the chromium mass balance (Fig. 2(b)), there is more Cr(VI) in substrate of no treatment, indicating microorganisms immobilize more of the Cr(VI) in the substrate by biosorption^17^. At the same time, the total content of Cr(III) in the substrate and supernatant is the highest in the untreated substrate test, confirming that the microorganism can achieve detoxification of Cr(VI) through biological reduction^18^.

### Phylotypes and diversity of microbial community

**Figure 3:**
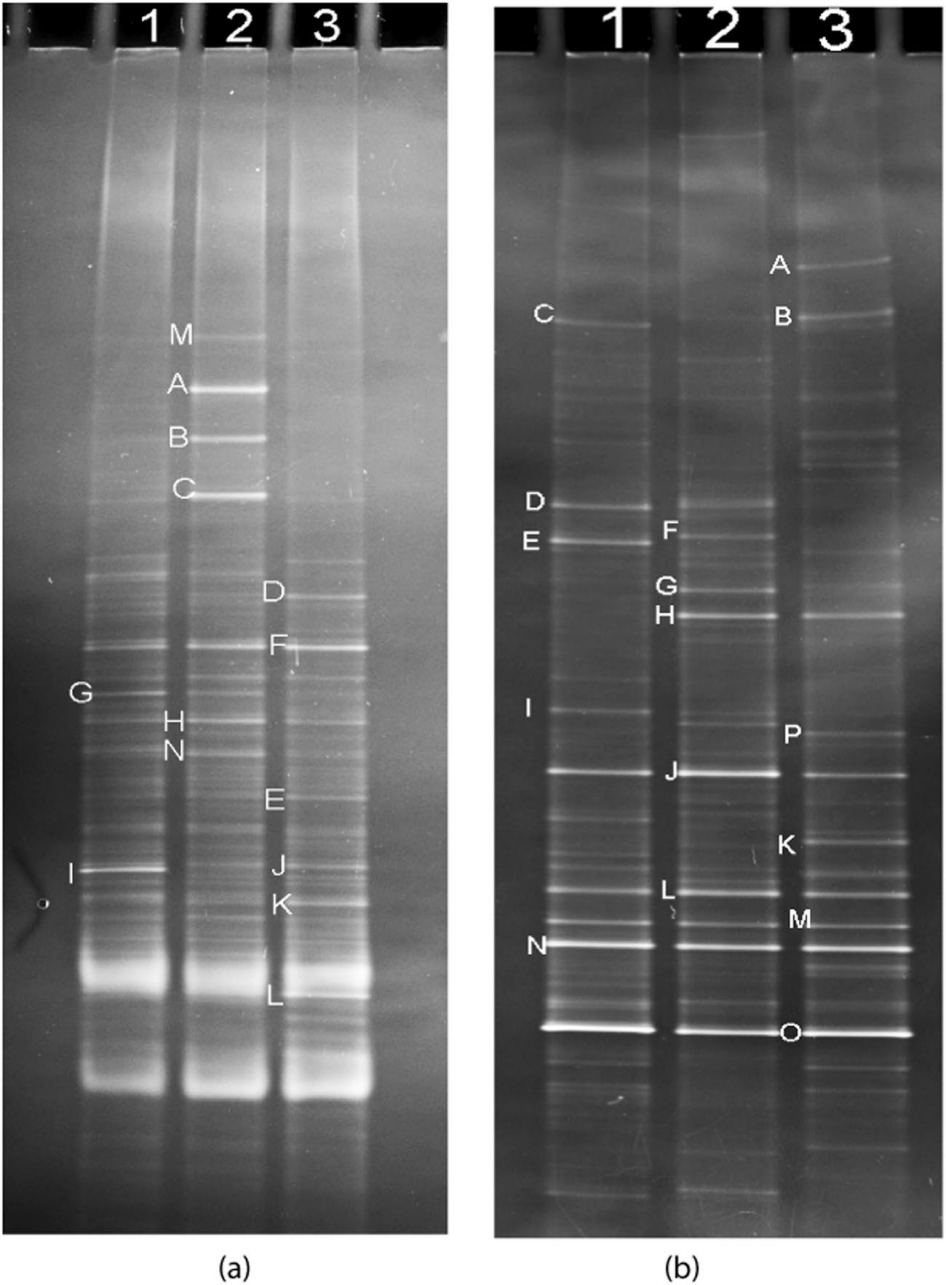
DGGE profiles of bacteria (**a**) and fungi (**b**) in substrates substrates of different CWs 1: 1# CWs; 2: 2# CWs; 3: 3# CWs.

Figure 3 shows the DGGE profiles of bacteria and fungi in substrates of different CWs. As shown in Fig. 3(a), there are significant differences in the number and population of bacteria in the three constructed wetland substrates. Firstly, the obvious bands number of the 2# CWs DGGE profile is the highest, indicating that under the stress of Cr(VI), the microbial community in the substrate is inhibited and the biodiversity is reduced. which may cause the substrate ecosystem to become fragile^32^. Comparing with the DGGE profiles of 1# CWs and 3# CWs, there is a smaller number of bands in 1# CWs DGGE profile, suggesting that under the stress of Cr(VI), the *Leersia hexandra Swartz* significantly affect the population of bacteria. The results of Di calculated by Shannon Wiener Index (H’), Simpson Index (D), and Evenness Index (J’) methods (Table 3) also confirm this point. Secondly, the bands of M, A, B, and C in 2# CWs DGGE profile do not appear in the other two DGGE profiles, while many new lanes are in the DGGE profiles of 1# CWs and 3# CWs. This shows the bacteria represented by bands M, A, B, and C are very sensitive to Cr(VI). Because of Cr(VI) stress, they may die or be dormant, and be rapidly replaced the Cr(VI)-tolerant bacteria. The lower values of similarity coefficients (Cs) from “Quantity One” software (Table 4) prove that the bacterial communities in the three samples have large differences. Thirdly, the dominant bacteria in the 2# CWs substrate are the species represented by bands of A, B, C and F, while the dominant bacteria in the 1# CWs and 3# CWs substrates are the species represented by bands of F, G and I and lanes F and K, respectively. For 1# CWs, the sequence and comparison results of the bacteria represented by bands F, G and I are listed in Table 5. They are directed to *Sphingomonas* sp., *Cystobacter* sp., and *Acidobacteria bacterium*, respectively.

**Table 3:** Diversity index of bacterium and fungus in different substrates of CWs.

Sample	Bacterium			Fungus		
	H′	D	J′	H′	D	J′
1 # CWs	2.059	0.7273	0.5703	2.523	0.8690	0.7745
2# CWs	2.333	0.8296	0.6283	2.813	0.9129	0.8046
3# CWs	2.213	0.7848	0.6177	2.842	0.9105	0.8276

**Table 4:** Similarity coefficients (Cs) of bacterium and fungus in different substrates of CWs.

Sample	Bacterium			Fungus		
	1	2	3	1	2	3
1 # CWs	100	67.5	65.2	100	61	62.6
2# CWs	67.5	100	71.4	61	100	61.5
3# CWs	65.2	71.4	100	62.6	61.5	100

**Table 5:** The similarities of 16S rDNA-V3 sequence and ITS1 sequence to to closest relatives of DNA recovered from the respective bands in DGGE profiles of 1# CWs.

Band No.	Closest relative and accession number	Sequence similarity (%)	Note
F	Sphingomonas sp. 1PNM-26 16S ribosomal RNA gene (JQ608334.2)	93%	bacteria
G	Cystobacter sp. 94032 16S ribosomal RNA gene (DQ520899.1)	86%	
I	Acidobacteria bacterium UCL-046 16S ribosomal RNA gene (JF707491.1)	90%	
C	Draparnaldioides simplex isolate 7Drp_sim7 internal transcribed spacer 1 and 5.8S ribosomal RNA gene (JX123273.1)	92%	fungus
D	Hydrodictyon reticulatum 18S rRNA gene (partial), 5.8S rRNA gene, 28S rRNA gene (partial), ITS1 and ITS2 (HE610123.1)	97%	
E	Fungal endophyte 18S rRNA gene (partial), ITS1, 5.8S rRNA gene, ITS2 and 28S rRNA gene (FN394699.1)	90%	
I	Gracilaria corticata var. cylindrica 18S ribosomal RNA gene (EU937777.1)	93%	
J	Scenedesmus sp. Ki4 genes for ITS1 (AB762691.1)	99%	
L	Desmodesmus sp. SGH-3 internal transcribed spacer 1 (KF689567.1)	91%	
M	Scenedesmus costatus genes for ITS1 (AB762692.1)	94%	
N	Desmodesmus sp. F51 internal transcribed spacer 1, 5.8S ribosomal RNA gene (JQ867370.1)	98%	
O	Hydrodictyon reticulatum 18S rRNA gene (HE610123.1)	99%	

From Fig. 3(b), it can be seen that the number of bands in the three DEEG profiles are basically the same, while the positions of the bands are very different. This indicates a significant change in the population of the fungus. The diversity index (Table 3) and similarity coefficients (Table 4) for fungus both prove this inference. The bands of J, L, M, N and O appear in the same location in all three DGGE profiles, indicating that the effects of Cr(VI) stress and rhizosphere exudates of the *Leersia hexandra Swartz* on them are not significant. The characteristic fungal flora bands are as follow: C, D, E and I for 1# CWs; F and G for 2# CWs; A, B, P and K for 3# CWs. The representative bands C, D, E, I, J, L, M, N, and O in the DEEG profile of 1# CWs were selected to amplify and identify the 18S rDNA V3 region gene of the fungus, and a series of measurements were performed. The Sequence comparison results are shown in Table 5. They mainly belong to *Sporotrichum*, and *Pellicularia species*.

### Identification of rhizosphere Cr(VI) reducing bacteria

**Figure 4:**
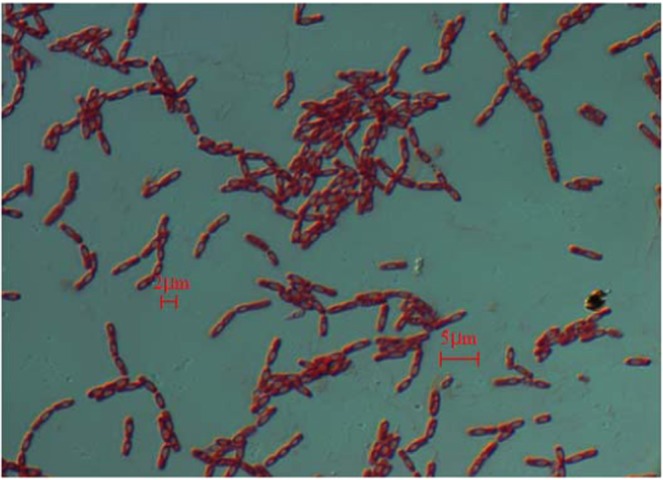
The micrograph of Cr(VI) reducing bacteria isolated from the rhizosphere of 1# CWs(GG).

**Figure 5:**
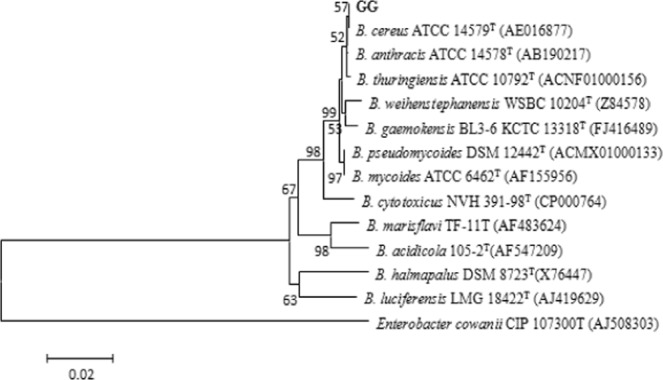
Phylogenetic tree of Cr(VI) reducing bacteria isolated from the rhizosphere of 1# CWs(GG).

The Cr(VI) reducing bacteria isolated from the rhizosphere of *Leersia hexandra Swartz*t are labeled as GG. Figure 4 and Table 6 show its cell morphology and physiological and biochemical characteristics, respectively. Compared with other studies^33,34^, the Cr(VI) reducing bacteria may be *Bacillus cereus*. Moreover, the phylogenetic tree of GG (Fig. 5) shows a significant genetic relationship with *Bacillus cereus*. Therefore, GG was identified as *Bacillus cereus*. It should be noted that DGGE profiles did not recover *Bacillus cereus* in the previous microbial community analysis. The reason may be that although *Bacillus cereus* is highly resistant to Cr(VI), it does not form a dominant flora in wetlands.

**Table 6:** The Physiological and Biochemical Characteristics of GG.

Characteristics	GG	Characteristics	GG	Characteristics	GG
Gram stain	+	Hydrolysis of starch	+	Catalase	+
Form of spore	+	Methyl red reaction	+	Casein hydrolysis	+
Arginine dihydrolase	+	Citric acid utilization	+	Voges-Proskauer test	+
Arginine dihydrolase	+	Gelatin hydrolysis	+	H_2_S generation	−
Lysine decarboxylase	−	Urease	−	Ornithine decarboxylase	−
Indole production	−	Negative control	−	Dextrin	+
D-maltose	w	D-trehalose	+	D-Cellobiose	−
Gentiobiose	−	Sucrose	w	Matsumoto	−
Stachyose	−	D- raffinose	−	α-D-lactose	−
D-melobiose	−	β- methyl -D-glucoside	w	D-salicin	w
N-acetyl-D-glucosamine	+	N-acetyl-β-D-mannosamine	−	N-acetyl-D-galactosamine	−
N-acetylneuraminic acid	−	1%, 4%, 8% NaCl	+	α-D-glucose	+
D-mannose	−	D-fructose	+	D-galactose	−
3-methyl-D-glucose	−	D-fucose	−	L-fucose	−
L-rhamnose	−	Inosine	−	1% sodium lactate	+
Fusidic acid	−	D-serine	+	D-sorbitol	−
D-mannitol	−	D-arabitol	−	Inositol	w
Glycerin	+	D-glucose-6-phosphate	+	D-fructose-6-phosphate	+
D-Aspartate	−	D-serine	+	Oleandoxin	−
Rifamycin SV	−	Dimethylamine tetracycline	−	Gelatin	+
Glycine-L-proline	−	L-Alanine	+	L-arginine	w
L-Aspartate	w	L-glutamic acid	w	L-histidine	+
L-pyroglutamic acid	w	L-serine	+	Lincomycin	−
Guanidine hydrochloride	+	Sodium tetradecanoate	−	Pectin	+
D-galacturonic acid	−	L-galactonolactone	−	D-Gluconic acid	+
D-glucuronic acid	w	Glucuronide	w	Mucic acid	−
Quinine	w	Saccharic acid	−	Vancomycin	−
Tetrazolium violet	−	Tetrazolium	−	p-Hydroxyphenylacetic acid	−
Methyl pyruvate	+	D-methyl lactate	−	L-lactic acid	+
Sodium bromate	w	α- ketoglutarate	w	D-malic acid	−
L-malic acid	w	Bromosuccinic acid	−	Nalidixic acid	w
Lithium chloride	+	Potassium citrate	+	γ- aminobutyric acid	−
α- hydroxybutyric acid	w	β-Hydroxy-D,L-butyric acid	−	α-butyric acid	w
Acetoacetate	+	Propionic acid	−	Acetic acid	w
Formic acid	+	Aztreonam	+	Sodium butyrate	+

Note: −, Negative; +, Positive; w, Weak reaction.

### Ability of *Bacillus cereus* to reduce Cr(VI)

#### Effect of pH on Cr(VI) removal

**Figure 6:**
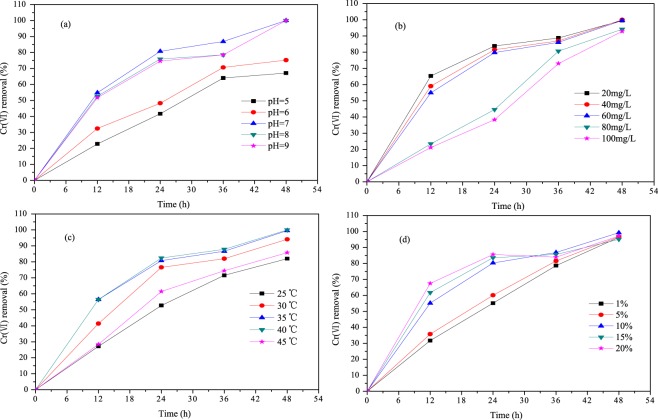
Effect of different pH (**a**), Cr(VI) initial concentrations (**b**), temperature (**c**), and *Bacillus cereus* inoculation (**d**) on Cr(VI) removal.

As Fig. 6(a) shown, *Bacillus cereus* has higher Cr(VI) removal rates under neutral and alkaline conditions, and the maximum removals were nearly 100% after 48 h. The reasons may be as follow: (1) under neutral and alkaline conditions, *Bacillus cereus* has stronger biological activity and cell metabolism which are benefit for Cr(VI) reduction^35^; (2) at low pH level, more hydrated ions are formed, causing protonation of negative sites on bacterial cells, which will reduce the amount of Cr(VI) biosorption^36^.

#### Effect of Cr(VI) concentration on Cr(VI) removal

It is obvious that with the increase of Cr(VI) concentration, the reduction effect of Cr(VI) by Bacillus cereus is decreasing (Fig. 6(b)). However, when the contact time is longer than 24 h, the influence of the Cr(VI) concentration becomes insignificant. The reasons may be as follows: (1) In the early stage, high concentration of Cr(VI) has a large negative effect on Bacillus cereus, such as oxidative stress, DNA and protein damage^37^; (2) After a period of time, Bacillus cereus is resistant to Cr(VI) stress and restores activity. This also indicates that Bacillus cereus has good adaptability to high concentrations of Cr(VI).

#### Effect of temperature on Cr(VI) removal

The effect of temperature on Cr(VI) removal is shown in Fig. 6(c). It is clear that when the temperature is 25 °C and 45 °C, Cr(VI) removals are lower. This may be attributed to the following reasons: (1) the Cr(VI) reductase activity of *Bacillus cereus* is lower when the temperature is higher or lower^38^; (2) When the temperature is in the range of 30–40 °C, the affinity of the Cr(VI) site or the binding site on the *Bacillus cereus* is higher^39^. In addition, the curves of 35 °C and 40 °C are almost coincident, indicating that the suitable temperature range for the reduction of Cr(VI) by *Bacillus cereus* is 35–40 °C.

#### Effect of *Bacillus cereus* inoculation on Cr(VI) removal

The results shown in Fig. 6(d) depict Cr(VI) removal during 48 h, when *Bacillus cereus* inoculation is increased from1.0 to 20%. When the time is less than 36 h, the removal rate of Cr(VI) increases with the increase of inoculation, which may be due to the increase of cell density of *Bacillus cereus*^35^. However, as the time is further extended, the difference in Cr(VI) removals is small. The reason may be that nutritional competition limits the reproduction of *Bacillus cereus*. This also indicates that *Bacillus cereus* has a strong reducing ability to Cr(VI).

## Conclusion

The microorganisms in substrates could improved Cr(VI) removal to 76.5%, and result in that more Cr(VI) was reduced to Cr(III). The dominant strains in the substrate of different CWs were *Sphingomonas* sp., *Cystobacter* sp., *Acidobacteria bacterium*, *Sporotrichum* and *Pellicularia species*. The rhizosphere Cr(VI) reducing bacteria of *Leersia hexandra Swartz* was identified as *Bacillus cereus*. And when the pH was 7, temperature was 35 °C, Cr(VI) concentration was less than 80 mg/L and inoculation was 10%, the removal rate of Cr(VI) by *Bacillus cereus* was close to 100%.

## References

[1] Dhal B, Thatoi HN, Das NN, Pandey BD (2013). Chemical and microbial remediation of hexavalent chromium from contaminated soil and mining/metallurgical solid waste: a review. J. Hazard. Mater..

[2] Sinha V, Manikandan NA, Pakshirajan K, Chaturvedi R (2017). Continuous removal of Cr(VI) from wastewater by phytoextraction using *Tradescantia pallida* plant based vertical subsurface flow constructed wetland system. Int. Biodeter. Biodegr..

[3] Zhang L, Fu FL, Tang B (2019). Adsorption and redox conversion behaviors of Cr(VI) on goethite/carbon microspheres and akaganeite/carbon microspheres composites. Chem. Eng. J..

[4] Sultana MY (2015). Integrated Cr(VI) removal using constructed wetlands and composting. J. Hazard. Mater..

[5] Madera-Parra CA, Peña MR, Peña EJ, Lens PNL (2015). Cr(VI) and COD removal from landfill leachate by polyculture constructed wetland at a pilot scale. Environ. Sci. Pollut. Res..

[6] Liu J (2014). Cr(VI) removal and detoxification in constructed wetlands planted with *Leersia hexandra Swartz*. Ecol. Eng..

[7] Liu J (2015). Function of *Leersia hexandra Swartz* in constructed wetlands for Cr(VI) decontamination: A comparative study of planted and unplanted mesocosms. Ecol. Eng..

[8] Zayed AM, Terry N (2003). Chromium in the environment: factors affecting biological remediation. Plant Soil.

[9] Maine MA, Hadad HR, Sánchez G, Caffaratti S, Pedro MC (2016). Kinetics of Cr(III) and Cr(VI) removal from water by two floating macrophytes. Int. J. of Phytoremediat..

[10] Zhang XH (2007). Chromium accumulation by the hyperaccumulator plant *Leersia hexandra Swartz*. Chemosphere.

[11] Charisse MD, Cagonoc MR, Vasquez J (2017). Enhanced chromium adsorption capacity via plasma modification of natural zeolites. Jpn. J. Appl. Phys..

[12] Zhang XL (2018). Enhanced removal performance of Cr(VI) by the core-shell zeolites/layered double hydroxides (LDHs) synthesized from different metal compounds in constructed rapid infiltration systems. Environ. Sci. Pollut. Res..

[13] Zhang XL, Zhang S, He F, Wu ZB (2007). Different performance of eight filter media in vertical flow constructed wetland: removal of organic matter, nitrogen and phosphorus. Fresenius Environ. Bull..

[14] Xu G, Lv YC, Sun JN, Shao HB, Wei LL (2012). Recent Advances in Biochar Applications in Agricultural Soils: Benefits and Environmental Implications. Clean-Soil, Air, Water.

[15] Kasak K (2018). Biochar enhances plant growth and nutrient removal in horizontal subsurface flow constructed wetlands. Sci. Total Environ..

[16] Tang XY (2017). Transformation of chlorpyrifos in integrated recirculating constructed wetlands (IRCWs) as revealed by compound-specific stable isotope (CSIA) and microbial community structure analysis. Bioresourc. Technol..

[17] Vendruscolo F, Ferreira GLDR, Filho NRA (2017). Biosorption of hexavalent chromium by microorganisms. Int. Biodeter. Biodegr..

[18] Huang YL, Feng H, Lu H, Zeng YH (2017). A thorough survey for Cr-resistant and/or -reducing bacteria identified comprehensive and pivotal taxa. Int. Biodeter. Biodegr..

[19] Singha A, Vyasb D, Malaviya P (2016). Two-stage phyto-microremediation of tannery effluent by *Spirodela polyrrhiza* (L.) Schleid. and chromium resistant bacteria. Bioresource Technol..

[20] Sultana MY, Akratos CS, Pavlou S, Vayenas DV (2014). Chromium removal in constructed wetlands: A review. Int. Biodeter. Biodegr..

[21] Michailides MK, Sultana MY, Tekerlekopoulou AG, Akratos CS, Vayenas DV (2013). Biological Cr(VI) removal using bio-filters and constructed wetlands. Water Sci. Techol..

[22] Dimitroula H (2015). Mitigation measures for chromium-VI contaminated groundwater-The role of endophytic bacteria in rhizofiltration. J. Hazard. Mater..

[23] Li H (2009). Comparisons of different hypervariable regions of *rrs* genes for fingerprinting of microbial communities in paddy soils. Soil Biol. Biochem..

[24] Thomas P, Sekhar AC (2017). Cultivation Versus Molecular Analysis of Banana (*Musa* sp.) Shoot-Tip Tissue Reveals Enormous Diversity of Normally Uncultivable Endophytic Bacteria. Microb. Ecol..

[25] Yiğittürk G, Uzel A (2015). Microbial community diversity associated with *Sarcotragus* sp. and *Petrosia ficiformis* from the Aegean Sea based on 16S rDNA-DGGE fingerprinting. Mar. Biol. Res..

[26] Ezeokoli OT, Gupta AK, Mienie C, Popoola TO, Bezuidenhout CC (2016). PCR-denaturing gradient gel electrophoresis analysis of microbial community in *soy-daddawa*, a Nigerian fermented soybean (*Glycine max* (L.) Merr.) condiment. Int. J. Food Microbiol..

[27] Chen GK (2018). Effects of influent loads on performance and microbial community dynamics of aerobic granular sludge treating piggery wastewater. J. Chem. Technol. Biotechnol..

[28] Murugavelh S, Mohanty K (2013). Isolation, identification and characterization of Cr(VI) reducing *Bacillus cereus* from chromium contaminated soil. Chem. Eng. J..

[29] Liu ZM, Wu Y, Lei CF, Liu PM, Gao MY (2012). Chromate reduction by a chromate-resistant bacterium, *Microbacterium* sp. World J. Microbiol. Biotechn..

[30] Wang L, Lin H, Dong YB, He YH, Liu CJ (2018). Isolation of vanadium-resistance endophytic bacterium PRE01 from *Pteris vittata* in stone coal smelting district and characterization for potential use in phytoremediation. J. Hazard. Mater..

[31] Ilyas S, Qamar MU, Rasool MH, Abdulhaq N, Nawaz Z (2016). Multidrug-resistant pathogens isolated from ready-to-eat salads available at a local market in Pakistan. Brit. Food J..

[32] Chen JH (2014). Heavy metal pollution decreases microbial abundance, diversity and activity within particle-size fractions of a paddy soil. FEMS Microbiol. Ecol..

[33] Fan JY (2012). Isolation, identification and characterization of a glyphosate-degrading bacterium, *Bacillus cereus* CB4, from soil. J. Gen. Appl. Microbiol..

[34] Li LH (2012). Screening and partial characterization of *Bacillus* with potential applications in biocontrol of cucumber *Fusarium* wilt. Crop Prot..

[35] Tripathi M, Garg SK (2014). Dechlorination of chloroorganics, decolorization, and simultaneous bioremediation of Cr^6+^ from real tannery effluent employing indigenous *Bacillus cereus* isolate. Environ. Sci. Pollut. Res..

[36] Shin MN (2012). Characterization of lead resistant endophytic *Bacillus* sp. MN3-4 and its potential for promoting lead accumulation in metal hyper accumulator *Alnus firma*. J. Hazard. Mater..

[37] Nayak AK, Panda SS, Basu A, Dhal NK (2018). Enhancement of toxic Cr(VI), Fe, and other heavy metals phytoremediation by the synergistic combination of native *Bacillus cereus* strain and *Vetiveria zizanioides* L. Int. J. Phytoremediat..

[38] Desai C, Jain K, Madamwar D (2008). Evaluation of *In vitro* Cr(VI) reduction potential in cytosolic extracts of three indigenous *Bacillus* sp. isolated from Cr(VI) polluted industrial landfill. Bioresourc. Technol..

[39] Sultan S, Mubashar K, Faisal M (2012). Uptake of toxic Cr(VI) by biomass of exo-polysaccharides producing bacterial strains. Afr. J. Microbiol. Res..

